# Variations in Using Diagnosis Codes for Defining Age-Related Macular Degeneration Cohorts

**DOI:** 10.3390/informatics11020028

**Published:** 2024-05-01

**Authors:** Fritz Gerald Paguiligan Kalaw, Jimmy S. Chen, Sally L. Baxter

**Affiliations:** 1 Division of Ophthalmology Informatics and Data Science, Viterbi Family Department of Ophthalmology and Shiley Eye Institute, University of California San Diego, 9415 Campus Point Dr MC0946, La Jolla, CA 92093, USA; 2 UCSD Health Department of Biomedical Informatics, University of California San Diego, 9415 Campus Point Dr MC0946, La Jolla, CA 92093, USA

**Keywords:** age-related macular degeneration, big data, data standards, electronic health records, informatics, international classification of diseases

## Abstract

Data harmonization is vital for secondary electronic health record data analysis, especially when combining data from multiple sources. Currently, there is a gap in knowledge as to how studies identify cohorts of patients with age-related macular degeneration (AMD), a leading cause of blindness. We hypothesize that there is variation in using medical condition codes to define cohorts of AMD patients that can lead to either the under- or overrepresentation of such cohorts. This study identified articles studying AMD using the International Classification of Diseases (ICD-9, ICD-9-CM, ICD-10, and ICD-10-CM). The data elements reviewed included the year of publication; dataset origin (Veterans Affairs, registry, national or commercial claims database, and institutional EHR); total number of subjects; and ICD codes used. A total of thirty-seven articles were reviewed. Six (16%) articles used cohort definitions from two ICD terminologies. The Medicare database was the most used dataset (14, 38%), and there was a noted increase in the use of other datasets in the last few years. We identified substantial variation in the use of ICD codes for AMD. For the studies that used ICD-10 terminologies, 7 (out of 9, 78%) defined the AMD codes correctly, whereas, for the studies that used ICD-9 and 9-CM terminologies, only 2 (out of 30, 7%) defined and utilized the appropriate AMD codes (*p* = 0.0001). Of the 43 cohort definitions used from 37 articles, 31 (72%) had missing or incomplete AMD codes used, and only 9 (21%) used the exact codes. Additionally, 13 articles (35%) captured ICD codes that were not within the scope of AMD diagnosis. Efforts to standardize data are needed to provide a reproducible research output.

## 1. Introduction

Age-related macular degeneration (AMD) is a progressive degenerative retinal disease that affects the macula and is one of the leading causes of blindness in the adult population in Western society aged 55 years and older [1]. Its development is multifactorial in origin, with a combination of different interactions between retinal microvasculature, metabolic, environmental, and genetic factors [1,2]. It has been classified by the Beckman Initiative for Macular Research Classification Committee into early, intermediate, and late AMD [3]. Late AMD has been subdivided into neovascular AMD or geographic atrophy. Neovascular AMD is characterized by the formation of new blood vessels within the macula, which may cause an accumulation of fluid or blood within the intraretinal, subretinal, or subretinal pigment epithelium (RPE) [4]. Geographic atrophy, on the other hand, is characterized by the appearance of atrophic lesions on the outer retina caused by the loss of photoreceptors and RPE [5]. Because of the complex nature of the disease process, numerous studies have emerged since it was first discovered to better understand the pathophysiology and management of such a common and yet blinding disease.

The widespread adoption of electronic health records (EHRs) has facilitated the availability of observational health data for clinical use or research. With this, several clinical registries in ophthalmology have been established and were noted to have grown significantly in the past decades, which could help in quality improvement and research [6]. Some examples of this are nationwide registries such as the American Academy of Ophthalmology Intelligent Research In Sight (IRIS^®^) Registry [7] and the National Institutes of Health (NIH) *All of Us* Research Program [8]. These data sources have integrated structured EHR data into large datasets that can be used for retrospective studies. For observational studies that entail a secondary analysis of EHR data, investigators often use standardized diagnosis codes to identify a cohort of patients relevant to their study question.

The World Health Organization established the International Classification of Diseases (ICD) as a standardized coding of human diseases from data reported globally. The clinical terms coded in the ICD are the main basis for recording diseases, which are used for health recording, statistics, and death certificates [9]. With several iterations, the ICD has been regularly updated throughout the years. The ICD-9 was initially published in 1977 and the ICD-10 in 1994. ICD-9 uses four to five digits to categorize specific diagnosis or pathology. In ICD-10, alphanumeric coding can reach as many as seven digits to provide further granularity of diagnosis. Additional provisions and modifications have been provided throughout the years [10]. In the United States, modifications of the ICD-9 and ICD-10 called Clinical Modifications (CMs) were developed to ensure the clinical accuracy and utility of disease codes [11]. Its latest revision (ICD-11) was adopted in 2019 and came into effect in early 2022, although the CM version for use in the United States has not yet been developed and widely implemented [12]. AMD diagnosis codes are available in ICD-9 and more extensively in ICD-10/ICD-10-CM (Table S1).

Despite the availability of the diagnosis codes for AMD, they may not necessarily be used consistently in observational studies involving EHR data. Lack of standardization in cohort definitions is a common challenge in observational research and can limit generalizability and reproducibility across studies if study cohorts are defined differently. Here, we conducted a review of observational studies using ICD codes to define cohorts of AMD patients to understand the current usage, variations, and opportunities for future improvement.

## 2. Methods

This study did not entail a direct analysis of health data and focused on reviewing published literature, which does not entail human subject research. It adhered to the tenets of the Declaration of Helsinki.

### 2.1. Article Search and Review

All articles published before the search (12 November 2023) were identified in PubMed using the following constructed terms in the search box: “macular degeneration AND (ICD OR diagnosis codes OR billing codes)”. Articles included in the Web of Science were also identified in the search box using the term “macular degeneration ICD”. The authors performed a manual review of each article, and the articles were included based on the following eligibility criteria: (1) Studies entailing analyses of retrospective data from electronic health records from clinical institutions, registries, or national or commercial claims databases; (2) used and listed diagnosis codes defined from ICD-9, ICD-9-CM, ICD-10, or ICD-10-CM; (3) provided the total number of subjects identified in the cohort of AMD codes used; and (4) full-text articles available in English. Articles that fulfilled the eligibility criteria were parsed, recorded, and analyzed.

### 2.2. Article Parsing

For each article that was included, the following data were extracted: study dataset (e.g., Veterans Affairs, registry, national or commercial claims database, and institutional EHR); year of publication; ICD terminology used; type of AMD the investigators aimed to study (e.g., all AMD patients, neovascular AMD, or non-neovascular AMD); the set of ICD codes the study investigators used to comprise their cohort definition; and the total number of subjects among the AMD cohort. For the purpose of comparison, diagnoses were categorized using synonymous terms. For example, “non-neovascular AMD” was used to encompass dry or non-exudative AMD, and “neovascular AMD” was used to include studies regarding wet or exudative AMD. The ICD codes were cross-checked for appropriateness with the diagnosis of AMD. For example, if the ICD codes included were related to neovascular AMD, non-neovascular AMD, or both (defined in our table as “AMD”).

### 2.3. Statistical Analysis

All data elements were tabulated, analyzed, and represented using Microsoft Excel and PowerPoint version 16.58 (Microsoft Corporation, Redmond, WA, USA). First, we analyzed the distribution of the data sources (e.g., Medicare, Veterans Affairs, institutional EHRs, etc.) by publication year. Next, we analyzed the extent of alignment between the codes used in each individual study against the set of relevant ICD codes for each terminology and cohort group. For example, to define neovascular AMD in ICD-9 terminology, the following code was deemed appropriate for the cohort definition: [36252]. If a study defined a cohort of neovascular AMD patients using ICD-9, we evaluated whether the set of codes they used for their cohort definition had an exact match with our gold standard cohort definition. If there was not an exact match, we evaluated whether there were too many codes included (such as including non-neovascular AMD codes or non-AMD codes entirely, for example) or too few codes included (such as not including some of the relevant codes for neovascular AMD). These were tabulated to calculate the proportion of studies with correct coding matches for each version of ICD terminology. See Supplemental Table S1 for a list of our standardized cohort definitions. We used Fisher’s exact test to evaluate whether there was a significant difference in the proportion of correctly matched cohort definitions between studies using the ICD-9 and ICD-10 terminologies. We also generated a Sankey diagram to illustrate the distribution of exact matches in codes, excess codes, and missing codes by ICD terminology. Finally, we conducted a bibliometric analysis of co-authorship networks and created visualizations of these networks using VOSViewer v1.6.20 (Centre for Science and Technology Studies, Leiden University, The Netherlands, www.vosviewer.com, accessed on 10 April 2024), a free software used for creating maps based on network data.

## 3. Results

**Figure 1.**
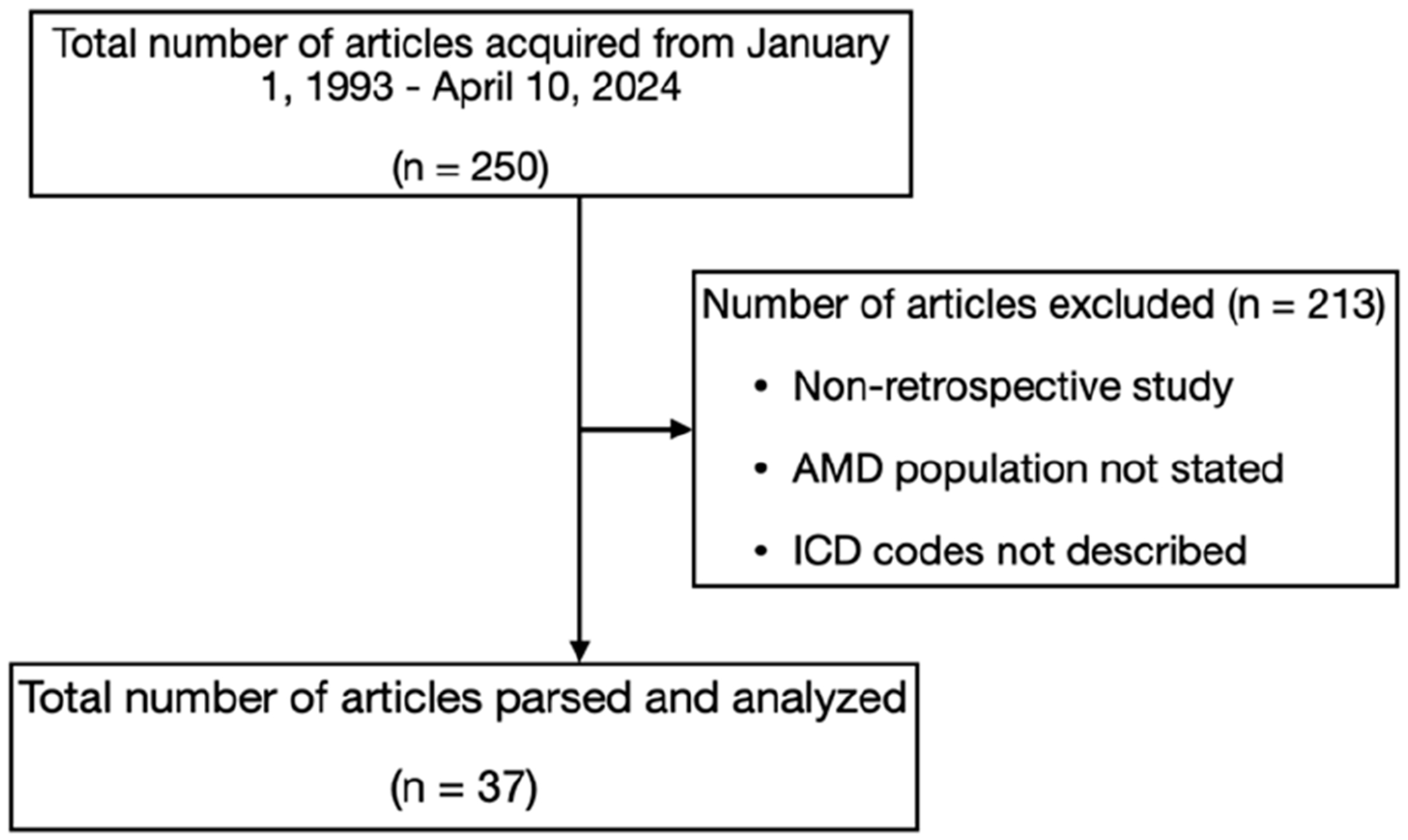
Flow diagram for obtaining articles from the PubMed and Web of Science search of observational studies that used electronic health record diagnosis codes in the ICD terminology system to define cohorts of patients with AMD. A total of 250 articles were acquired, 213 of which were excluded based on the eligibility criteria, leading to 37 articles left for parsing and analysis. ICD—International Classification of Diseases; AMD—age-related macular degeneration.

The initial query of PubMed and Web of Science yielded 250 articles. Two hundred and thirteen articles did not meet the eligibility criteria; hence, 37 articles were parsed and analyzed (Figure 1).

**Figure 2.**
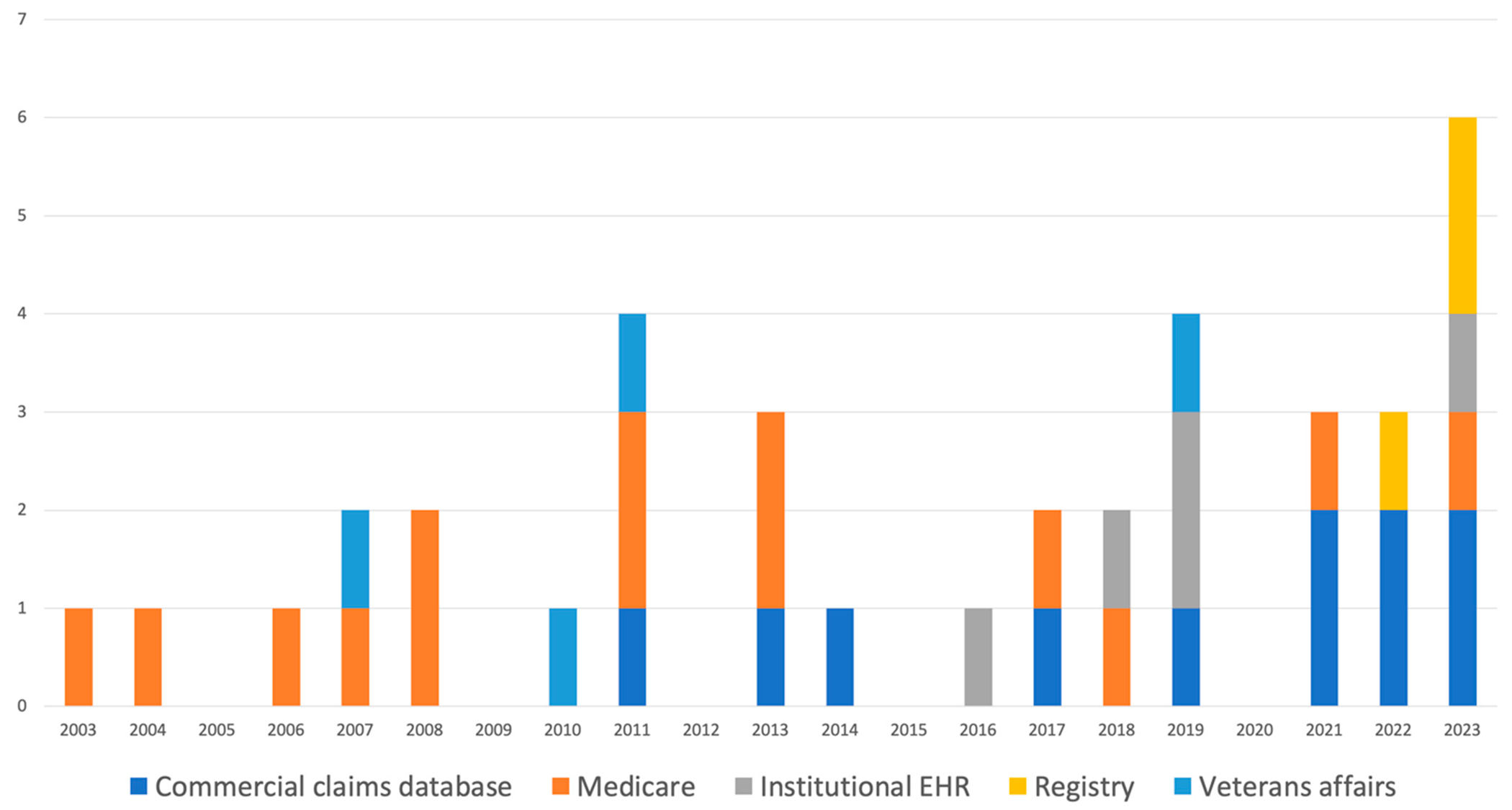
Stacked bar graph of dataset origins for articles per year. Each bar represents the total number of articles published that year, with individual-colored segments representing the various dataset origins. Note the consistent utilization of the Medicare database (orange) within the past two decades, with a gradual rise in the use of institutional EHRs (gray) within the last few years and the availability of various dataset origins in the last year.

A total of 8,398,072 subjects were studied among the eligible articles. Article publications ranged from 2003 to 2023, with the majority (22/37, 59%) published within the last decade. The largest proportion of the studies obtained their cohort from national claims databases (Medicare) (14, 38%). This was followed by commercial claims databases (9, 24%). Table 1 and Figure 2 show the distribution of dataset origin per year, showing the consistency of using the Medicare database within the past two decades and a rise in the use of institutional EHRs within the last few years, as well as the availability of published data using various dataset origins in the last year.

### EHR—Electronic Health Record

**Figure 3.**
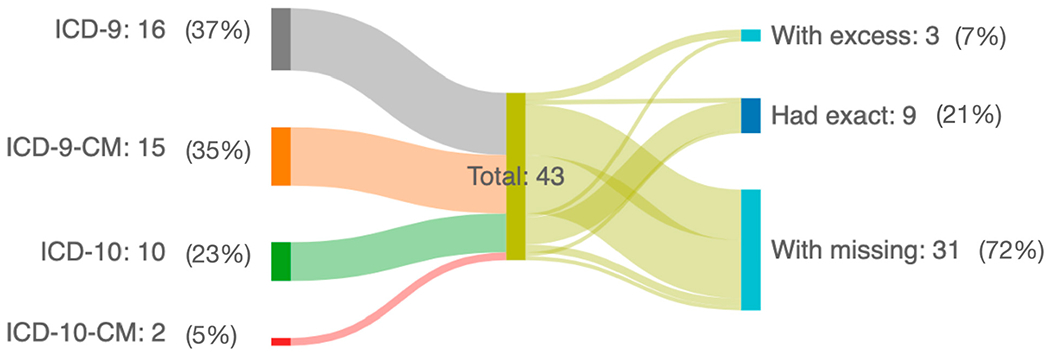
Sankey diagram showing the summary utilization of AMD codes per article. The study included 43 ICD codes from 37 articles; 6 articles used a combination of either ICD-9, ICD-9-CM, ICD-10, or ICD-10-CM codes. *With excess*—articles that used ICD codes other than those defined in Table S1, *Had exact*—articles that used the exact ICD codes defined in Table S1, and *With missing*—articles that had missing ICD codes defined in Table S1. AMD—age-related macular degeneration; ICD—International Classification of Diseases. This diagram was produced using an open-source tool, SankeyMATIC (www.sankeymatic.com, accessed on 10 April 2024).

Table 2 presents the AMD cohort definition used for each article, while Figure 3 summarizes how well the AMD cohort definitions align with the set of appropriate codes for the cohort of interest. Six (16%) articles used cohort definitions from two ICD terminologies. ICD-9 and ICD-9-CM were used in 12 (32%) and 13 (35%) articles, respectively, whereas ICD-10 and ICD-10-CM were used in 5 (14%) and 1 (3%) article, respectively, and combined ICD-9 and ICD-10 in 4 articles (11%). For the studies that used ICD-9 and 9-CM terminologies, only 2 (out of 30, 7%) defined and utilized the appropriate four AMD codes (362.5, 362.50, 362.51, and 362.52), on average missing two AMD codes per article. Most of the missing codes were either 362.5 or 362.50 for ICD-9/9-CM. For the studies that used ICD-10 terminologies, seven (out of nine, 78%) defined the AMD codes correctly (H35.3), while two used a different coding (H35.31 and H35.32). Based on our review, only two studies used ICD-10-CM terminologies; one defined all diagnosis AMD codes, and the other did not. Using Fisher’s exact test, our analysis showed that studies using ICD-10 terminology were significantly more likely to have an exact match with the appropriate set of codes compared to those using ICD-9 terminology (*p* = 0.0001).

Moreover, 13 articles included ICD codes that were outside the scope of the diagnosis of AMD (Table 3). These included diagnoses such as cystoid macular degeneration of the retina, drusen of the retina, serous detachment of the retinal pigment epithelium, and hemorrhagic detachment of the retinal pigment epithelium.

**Figure 4.**
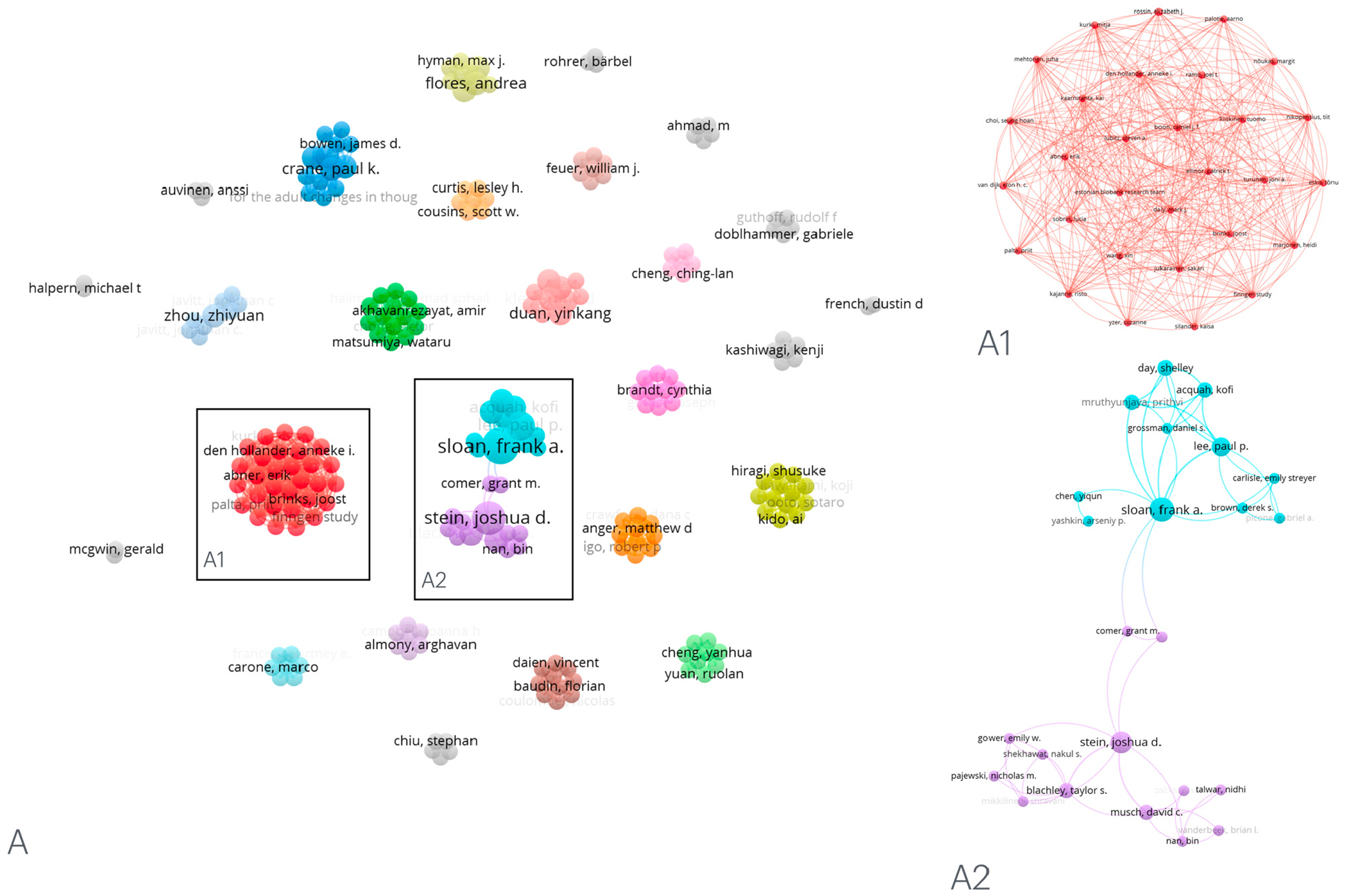
Network visualization of co-authors for original articles that used ICD-9, ICD-9-CM, ICD-10, and ICD-10-CM to define AMD. Overall, 27 clustered groups used AMD cohort definitions from different ICD terminologies (**A**). Inset (**A1**) shows the co-authorship network in one cluster in a single group, and inset (**A2**) shows a network of co-authors with other groups known as links. AMD—age-related macular degeneration; ICD—International Classification of Diseases.

We used VOSViewer to map co-author networks, as shown in Figure 4. This co-authorship analysis refers to the relatedness or link of items based on the number of co-authored documents. We used the co-authorship network to determine the group of co-authors and the links between these co-authors who studied AMD using controlled terminologies such as ICD. Our analysis revealed that authors clustered differently based on the cohort definitions of AMD, using ICD-9, ICD-9-CM, ICD-10, and ICD-10-CM. Out of the 37 reviewed articles, we found 27 clustered groups that used cohort definitions from different ICD terminologies.

## 4. Discussion

The present study systematically reviewed 37 published articles that used different definitions of AMD based on ICD-9 and 10 terminologies in defining cohorts for their studies. The present study uncovered the following findings: (1) The use of national databases serves as an important tool to extract big data, with institutional EHRs becoming increasingly used in the last few years to capture patient data and relevant information; (2) There has been underutilization of AMD diagnosis codes, which may lead to underestimating a set of cohorts; and (3) The use of non-AMD diagnosis codes, which may lead to overestimation of a set of cohorts.

The *first revision* of the ICD (ICD-1) was established over a century ago and has been on periodic revision thus far. The *ninth* and *tenth* revisions (ICD-9 and ICD-10) have been implemented since 1979 and 1999, respectively [50]. Medicare is a federal health insurance program generally for individuals over 65 years of age among US citizens [51] and has over 65 million beneficiaries as of March 2023 [52]. Studies using Medicare administrative claims were first published in 1979 and have since been growing [51]. Since AMD commonly affects the older adult population, using Medicare claims would be advantageous to use for studying AMD. This was reflected in our review, as Medicare databases had the highest proportion among the observational studies reviewed. Although national registries and commercial claims-based data provide heterogeneous and robust patient data, limitations such as generalizability, coverage restrictions, lack of billing codes, difficulty accessing and using the data, and understanding the data may deter sampling methods [53–56]. The passage of the Health Information Technology for Economic and Clinical Health (HITECH) Act of 2009 paved the way to advancing EHR use [57]. One of its potential advantages is the improved ability to conduct research and ease of access [58]. In ophthalmology, one advantage to using EHR data is the availability of specialty-specific information that can be linked and integrated into the patient data, such as multimodal retinal imaging data like fundus images, optical coherence tomographic scans, and visual fields. The usage of institutional EHR data in studying AMD has also been increasing, and as seen from our review, it has been notable within the past decade.

The second key finding of the present study was the underutilization of AMD diagnosis codes. ICD-9-CM has four AMD condition codes, with 362.5 (degeneration of macula and posterior pole) and 362.50 (macular degeneration [senile], unspecified) being distinct from each other but can be mistaken as one due to a minor addition (the fifth digit: number 0). This can confuse clinicians or investigators when inputting codes and can underestimate the cohort when doing research. The other two (362.51 [nonexudative senile macular degeneration] and 362.52 [exudative senile macular degeneration]) have been the most commonly used codes in each cohort. In studies where “AMD” was the target cohort, the studies averaged two unused codes, which may underrepresent the population. A recent study on AMD condition coding reported an underreporting of geographic atrophy, an advanced form of AMD, due to incorrect coding as intermediate dry AMD from the seventh digit of the ICD-10-CM coding [59]. Regarding the use of ICD-10, which only provides a single code for AMD (H35.3—degeneration of macular and posterior pole), nearly all studies captured the proper code. The ICD-10-CM coding for AMD has become more specific, adding subclassifications to the disease classification [60]. It has more data granularity, including laterality, disease classification, and clinical activity (Table S1). In terms of the use of ICD-10-CM terminology, one study identified 16 codes for neovascular AMD, and the other only targeted 2 out of the 47 codes for AMD in general. However, transitioning from aggregate (ICD-9-CM) to granular (ICD-10-CM) data poses some challenges. The complexity of coding makes it difficult for physicians to participate in encoding to ensure an appropriate diagnosis [61]. As seen from the present results, most studies used a number of codes, less than what is available, to define the AMD cohort, which may lead to underrepresenting the targeted population. Our diagram illustrates these, where only 21% had utilized the correct diagnosis codes.

The third key finding was using non-AMD diagnosis codes from the ICD terminologies. Thirteen (35%) of the reviewed articles were noted to have additional codes unrelated to the diagnosis of AMD, even though the stated patient population of interest was AMD. This included the following: serous detachment of the retinal pigment epithelium (362.42), hemorrhagic detachment of the retinal pigment epithelium (362.43), cystoid macular degeneration of the retina (362.53), and drusen (degenerative) of the retina (362.57). Although the first three diagnoses can be a consequence of AMD, these diagnoses are not specific to AMD. Including the codes may dilute the target population and may even inadvertently include other primary causes of such diagnoses. The clinical hallmark of non-neovascular AMD is drusen, which are yellowish deposits at the level of the retinal pigment epithelium [62]. According to the clinical classification of AMD [3], early AMD is considered when drusen with a size of >63 μm and ≤125 μm is apparent. Drusen alone is not considered a class of AMD since normal aging changes can present with druse [3]. Nine out of thirteen of the articles incorporated drusen (362.57) as an inclusion to define their AMD cohort, which may again dilute the results since the prevalence of drusen can be as high as 91% in the normal population [63].

In the field of ophthalmology, specifically vitreoretinal diseases, improving the standardized representation of diseases is ongoing. A recent report by Kalaw and colleagues [64] discovered several important retinal diagnoses not represented in the Systematized Nomenclature of Medicine (SNOMED). In one of the articles reviewed in this study [28], polypoidal choroidal vasculopathy, considered a pachychoroid disorder, and idiopathic choroidal neovascularization were defined as AMD, even though these diagnoses warrant a separate coding system due to the nature of the disorder and distinct pathophysiology. A study by Tavakoli and colleagues [65] reported that some ophthalmic infectious and traumatic diagnoses do not accurately match the ICD-10-CM diagnosis and are considered a wide match. Lastly, in a study by Cai and colleagues [66], there were noted gaps in diagnosis codes and eye exam data elements. Future collaborative studies may be needed to supply the missing elements and concepts in ophthalmology.

The present study has limitations. It obtained peer-reviewed articles from PubMed and Web of Science. Other biomedical literature databases, such as Google Scholar or Scopus, may provide more relevant articles. Additionally, the study focused on variations in the use of ICD terminologies. Additional variations may be present when using SNOMED or other standardized terminologies.

## 5. Conclusions

In summary, there is substantial variation in the use of ICD diagnosis codes for identifying cohorts of AMD subjects, with possible implications of under-sampling, oversampling, and a lack of reproducibility across studies. This could affect the ongoing efforts in understanding and treating one of the most common diseases in the field of ophthalmology. Awareness among healthcare professionals, especially ophthalmologists, with the appropriate and specific codes should be practiced. Standardization of cohort definitions should be observed to provide reproducible results.

## Supplementary Material

supplementarytables

## Figures and Tables

**Table 1.** Characteristics of articles with AMD cohort definitions using ICD codes review, spanning studies published between 2003 and 2023.

Author	Year of Publication	Dataset Origin	Number of Patients Included
Duan et al. [13]	2003	Medicare	167,034
Sloan et al. [14]	2004	Medicare	4,280
Halpern et al. [15]	2006	Medicare	58,594
Zlateva et al. [16]	2007	Medicare	26,057
Swanson et al. [17]	2007	Veterans Affairs	614
Liao et al. [18]	2008	Medicare	137,838
Day et al. [19]	2008	Medicare	20,671
Latkany et al. [20]	2010	Veterans Affairs	226
Day et al. [21]	2011	Medicare	12,465
Stein et al. [22]	2011	Claims database	2,252,515
French et al. [23]	2011	Veterans Affairs	3,021
Stein et al. [24]	2011	Medicare	23,941
Stein et al. [25]	2013	Commercial Claims database	103
Sloan et al. [26]	2013	Medicare	2,151
Qualls et al. [27]	2013	Medicare	23,133
Kume et al. [28]	2014	Commercial Claims database	3,058
Leisy et al. [29]	2016	Institutional EHR	107
Lee et al. [30]	2017	Commercial Claims database	933
Gower et al. [31]	2017	Medicare	195,812
Chiu et al. [32]	2018	Institutional EHR	579
Rosenfeld et al. [33]	2018	Medicare	3,462,402
Halladay et al. [34]	2019	Veterans Affairs	504,027
Schnabolk et al. [35]	2019	Commercial Claims database	37,252
Lee et al. [36]	2019	Institutional EHR	273
Lee et al. [37]	2019	Institutional EHR	1,036
Almony et al. [38]	2021	Commercial Claims database	6,076
Hwang et al. [39]	2021	Medicare	668
Nestler et al. [40]	2021	Commercial Claims database	1,000
Loukovaara et al. [41]	2022	Registry	2,947
Creuzot-Garcher et al. [42]	2022	Commercial Claims database	432,961
Kido et al. [43]	2022	Commercial Claims database	246,064
Matsumiya et al. [44]	2023	Institutional EHR	1,913
Liu et al. [45]	2023	Registry	6,157
Moi et al. [46]	2023	Commercial Claims database	312,404
Rämö et al. [47]	2023	Registry	8,913
Javitt et al. [48]	2023	Medicare	25,820
Moir et al. [49]	2023	Commercial Claims database	415,027

AMID—age-related macular degeneration; EHR—electronic health record.

**Table 2.** Characteristics of articles according to AMD cohort definition and ICD terminologies and codes used.

Article Author	AMD Cohort of Interest	ICD Terminology Used	ICD Codes Used	Correct Codes Used	Missing Codes
Duan et al. [13]	AMD	9	362.42, 362.43, 362.52, 362.53, 362.5, 362.50, 362.51, 362.57	362.52, 362.5, 362.50, 362.51	
Sloan et al. [14]	AMD	9	362.51, 362.57, 362.52, 362.53, 362.5, 362.50	362.51, 362.52, 362.5, 362.50	
Swanson et al. [17]	AMD	9	362.50, 362.51, 362.52	362.50, 362.51, 362.52	362.5
Day et al. [19]	AMD	9	362.50, 362.52, 362.51, 362.57	362.50, 362.51, 362.52	362.5
Leisy et al. [29]	AMD	9	362.5, 362.51, 362.52	362.5, 362.51, 362.52	362.5
Chiu et al. [32]	AMD	9	362.50, 362.51, 362.52	362.50, 362.51, 362.52	362.5
Rosenfeld et al. [33]	AMD	9	362.50, 362.51, 362.52	362.50, 362.51, 362.52	362.5
Schnabolk et al. [35]	AMD	9	362.50, 362.51, 362.52	362.50, 362.51, 362.52	362.5
Lee et al. [36]	AMD	9	3625A, 3625B	362.5	362.50, 362.51, 362.52
Lee et al. [37]	AMD	9	362.50, 362.51, 362.52	362.50, 362.51, 362.52	362.5
Hwang et al. [39]	AMD	9	3625A, 3625B	362.5	362.50, 362.51, 362.52
Liu et al. [45]	neovascular AMD	9	362.52, 362.42, 362.43	362.52	362.5, 362.50, 362.51
Moi et al. [46]	neovascular AMD	9	362.52	362.52	362.5, 362.50, 32.51
Rämö et al. [47]	neovascular AMD	9	362.52, 362.42, 362.43	362.52	362.5, 362.50, 362.51
Javitt et al. [48]	non-neovascular AMD	9	362.51, 362.57	362.51	362.5, 362.50, 362.52
Moir et al. [49]	AMD	9	362.50, 362.51, 362.52	362.50, 362.51, 362.52	
Kume et al. [28]	AMD	10	H35.3	H35.3	
Nestler et al. [40]	AMD	10	H35.3	H35.3	
Loukovaara et al. [41]	AMD	10	H35.30	H35.3	
Creuzot-Garcher et al. [42]	AMD	10	H35.31, H35.32		H35.3
Kido et al. [43]	AMD	10	H35.30	H35.3	
Matsumiya et al. [44]	neovascular AMD	10	H353	H35.3	
Liu et al. [45]	neovascular AMD	10	H35.3	H35.3	
Moi et al. [46]	neovascular AMD	10	H35.3	H35.3	
Rämö et al. [47]	non-neovascular AMD	10	H35.32		H35.3
Moir et al. [49]	AMD	10	H35.30, H35.31, H35.32	H35.3	
Halladay et al. [34]	AMD	10-CM	H35.31, H35.32	H35.31, H35.32	H35.30, H35.311, H35.3110, H35.3111, H35.3112, H35.3113, H35.3114, H35.312, H35.3120, H35.3121, H35.3122, H35.3123, H35.3124, H35.313, H35.3130, H35.3131, H35.3132, H35.3133, H35.3134, H35.319, H35.3190, H35.3191, H35.3192, H35.3193, H35.3194, H35.321, H35.3210, H35.3211, H35.3212, H35.3213, H35.322, H35.3220, H35.3221, H35.3222, H35.3223, H35.323, H35.3233, H35.35.329, H35.3290, H35.3292, H35.3293
Almony et al. [38]	neovascular AMD	10-CM	H35.3210, H35.3211, H35.3212, H35.3213, H35.3220, H35.3221, H35.3222, H35.3223, H35.3230, H35.3231, H35.3232, H35.3233, H35.3290, H35.3291, H35.3292, H35.3293	H35.3210, H35.3211, H35.3212, H35.3213, H35.3220, H35.3221, H35.3222, H35.3223, H35.3230, H35.3231, H35.3232, H35.3233, H35.3290, H35.3291, H35.3292, H35.3293	
Halpern et al. [15]	AMD	9-CM	362.51, 362.52, 362.57	362.51, 362.52	362.5, 362.50
Zlateva et al. [16]	AMD	9-CM	362.42-362.43, 362.52, 362.53, 362.5, 362.50, 362.51, 362.57	362.5, 362.51, 362.52	362.5, 362.50
Liao et al. [18]	AMD	9-CM	362.50, 362.51, 362.57, 362.52	362.50, 362.51, 362.52	362.5
Latkany et al. [20]	AMD	9-CM	362.51-362.52	362.51, 362.52	362.5, 362.50
Day et al. [21]	AMD	9-CM	362.50-52, 362.57	362.51, 362.52	362.5
Stein et al. [22]	AMD	9-CM	362.51, 362.52	362.51, 362.52	362.5, 362.50
French et al. [23]	AMD	9-CM	362.51, 362.52, 362.5, 362.50, 362.53, 362.57	362.5, 362.51, 362.52	362.5
Stein et al. [24]	neovascular AMD	9-CM	362.42, 362.43, or 362.52	362.52	362.5, 362.50, 362.51
Stein et al. [25]	neovascular AMD	9-CM	362.52	362.52	362.5, 362.50, 362.53
Sloan et al. [26]	neovascular AMD	9-CM	362.52, 362.42, 362.43	362.52	362.5, 362.50, 362.51
Qualls et al. [27]	AMD	9-CM	362.52	362.52	362.5, 362.50, 362.51
Lee et al. [30]	neovascular AMD	9-CM	362.52	362.52	362.5, 362.50, 362.51
Gower et al. [31]	neovascular AMD	9-CM	362.52	362.52	362.5, 362.50, 362.51
Halladay et al. [34]	non-neovascular AMD	9-CM	362.50, 362.51, 362.57	362.5, 362.51	362.50, 362.52
Matsumiya et al. [44]	non-neovascular AMD	9-CM	362.52	362.52	362.5, 362.50, 362.51

By definition, non-neovascular AMD is synonymous with dry AMD, and neovascular AMD is synonymous with wet AMD. AMD—age-related macular degeneration; ICD—International Classification of Diseases; CMs—clinical modifications.

**Table 3.** Examples of non-AMD diagnoses of additional ICD codes included in select articles.

Article Number	Additional Codes	Codified Diagnosis
	362.53	Cyatoid macular degeneration of retina
Duan et al. [13]	362.57	Drusen (degenerative) of retina
	362.42	Serous detachment of retinal pigment epithelium
	362.43	Hemorrhagic detachment of retinal pigment epithelium

Sloan et al. [14]	362.53	Cystoid macular degeaeration of retina
	362.57	Drusen (degenerative) of retina

Halpern et al. [15]	362.57	Drusen (degenerative) of retina

Zlateva et al. [16]	362.42	Serous deiachment of retinal pigment epithelium
	362.43	Hemorrhagic detachment of retinal pigment epithelium

	362.42	Serous detachment of retinal pigment epithelium
Liao et al. [18]	362.43	Hemorrhagic datachment of retinal pigment epithelium
	362.53	Cystoid macular degeneration of retina
	362.57	Drusen (degenerative) of retina

Day et al. [19]	362.43	Hemorrhagic detachment of retinal pigment epithelium

Day et al. [21]	362.42	Serous detachment of retinal pigment epithelium
	362.43	Hemorrhagic detachment of retinal pigment epithelium

Stein et al. [22]	362.57	Drusen (degenerative) of retina

Stein et al. [25]	362.57	Drusen (degenerative) of retina

Sloan et al. [26]	362.53	Cystoid macular degeneration of retina
	362.57	Drusen (degenerative) of retina

Lee et al. [30]	362.57	Drusen (degenerative) of retina

Chiu et al. [32]	362.57	Drusen (degenerative) of retina

Javitt et al. [48]	362.42	Serous detachment of retinal pigment epithelium
	362.43	Hemorrhagic detachment of retinal pigment epithelium

AMD—age-related macular degeneration; ICD—International Classification of Diseases.

## Data Availability

Not applicable.
