# Supplementary material for: Variations in Using Diagnosis Codes for Defining Age-Related Macular Degeneration Cohorts
Source: Informatics (MDPI). Author manuscript; Available in PMC 2025 Feb 26. (PMC11864795; doi:10.3390/informatics11020028)
Supplement: supplementarytables [file NIHMS2009073-supplement-supplementarytables.zip › informatics-2821161-supplementary.pdf]

Table S1. Concept codes from four International Classification of Diseases Terminologies

ICD Terminology	Code
ICD-9	
Macular degeneration (senile), unspecified	36250
Nonexudative senile macular degeneration	36251
Exudative senile macular degeneration	36252
ICD-10	
Degeneration of macula and posterior pole	H35.3
Senile macular degeneration (atrophic) (exudative)	
ICD-9-CM	
Degeneration of macula and posterior pole	362.5
Macular degeneration (senile), unspecified	362.50
Nonexudative senile macular degeneration	362.51
Exudative senile macular degeneration	362.52
ICD-10-CM	
Unspecified macular degeneration	H35.30
Nonexudative age-related macular degeneration	H35.31
Nonexudative age-related macular degeneration, right eye	H35.311
Nonexudative age-related macular degeneration, right eye, stage unspecified	H35.3110
Nonexudative age-related macular degeneration, right eye, early dry stage	H35.3111
Nonexudative age-related macular degeneration, right eye, intermediate dry stage	H35.3112
Nonexudative age-related macular degeneration, right eye, advanced atrophic without subfoveal involvement	H35.3113
Nonexudative age-related macular degeneration, right eye, advanced atrophic with subfoveal involvement	H35.3114
Nonexudative age-related macular degeneration, left eye	H35.312
Nonexudative age-related macular degeneration, left eye, stage unspecified	H35.3120
Nonexudative age-related macular degeneration, left eye, early dry stage	H35.3121
Nonexudative age-related macular degeneration, left eye, intermediate dry stage	H35.3122
Nonexudative age-related macular degeneration, left eye, advanced atrophic without subfoveal involvement	H35.3123

Nonexudative age-related macular degeneration, left eye, advanced atrophic with subfoveal involvement	H35.3124
Nonexudative age-related macular degeneration, bilateral	H35.313
Non-exudative age-related macular degeneration, bilateral, stage unspecified	H35.3130
Nonexudative age-related macular degeneration, bilateral, early dry stage	H35.3131
Nonexudative age-related macular degeneration, bilateral, intermediate dry stage	H35.3132
Nonexudative age-related macular degeneration, bilateral, advanced atrophic without subfoveal involvement	H35.3133
Nonexudative age-related macular degeneration, bilateral, advanced atrophic with subfoveal involvement	H35.3134
Nonexudative age-related macular degeneration, unspecified eye	H35.319
Non-exudative age-related macular degeneration, unspecified eye, stage unspecified	H35.3190
Nonexudative age-related macular degeneration, unspecified eye, early dry stage	H35.3191
Nonexudative age-related macular degeneration, unspecified eye, intermediate dry stage	H35.3192
Nonexudative age-related macular degeneration, unspecified eye, advanced atrophic without subfoveal involvement	H35.3193
Nonexudative age-related macular degeneration, unspecified eye, advanced atrophic with subfoveal involvement	H35.3194
Exudative age-related macular degeneration	H35.32
Exudative age-related macular degeneration, right eye	H35.321
Exudative age-related macular degeneration, right eye, stage unspecified	H35.3210
Exudative age-related macular degeneration, right eye, with active choroidal neovascularization	H35.3211
Exudative age-related macular degeneration, right eye, with inactive choroidal neovascularization	H35.3212
Exudative age-related macular degeneration, right eye, with inactive scar	H35.3213
Exudative age-related macular degeneration, left eye	H35.322
Exudative age-related macular degeneration, left eye, stage unspecified	H35.3220
Exudative age-related macular degeneration, left eye, with active choroidal neovascularization	H35.3221
Exudative age-related macular degeneration, left eye, with inactive choroidal neovascularization	H35.3222
Exudative age-related macular degeneration, left eye, with inactive scar	H35.3223
Exudative age-related macular degeneration, bilateral	H35.323
Exudative age-related macular degeneration, bilateral, stage unspecified	H35.3230
Exudative age-related macular degeneration, bilateral, with active choroidal neovascularization	H35.3231
Exudative age-related macular degeneration, bilateral, with inactive choroidal neovascularization	H35.3232

Exudative age-related macular degeneration, bilateral, with inactive scar	H35.3233
Exudative age-related macular degeneration, unspecified eye	H35.329
Exudative age-related macular degeneration, unspecified eye, stage unspecified	H35.3290
Exudative age-related macular degeneration, unspecified eye, with active choroidal neovascularization	H35.3291
Exudative age-related macular degeneration, unspecified eye, with inactive choroidal neovascularization	H35.3292
Exudative age-related macular degeneration, unspecified eye, with inactive scar	H35.3293

ICD – International Classification of Diseases, CM – Clinical Modifications
